# Circ_072697 knockdown promotes advanced glycation end products-induced cell proliferation and migration in HaCaT cells via miR-3150a-3p/KDM2A axis

**DOI:** 10.1186/s12902-023-01430-2

**Published:** 2023-09-19

**Authors:** Ming Tian, Jiajun Tang, Rong Huang, Jiaoyun Dong, Huiying Jia

**Affiliations:** 1 Department of Burn, Wound Healing Center, Shanghai Burn Institute, Ruijin Hospital, Shanghai Jiao Tong University School of Medicine, Shanghai, 200025 China; 2 Wound Healing Center, Department of Burn, Ruijin Hospital, Shanghai Jiao Tong University School of Medicine, Shanghai, 200025 China; 3 Department of General Surgery, Putuo Hospital Shanghai University of Traditional Chinese Medicine, Shanghai, 200062 China; 4 Department of Endocrine and Metabolic Diseases, Shanghai Institute of Endocrine and Metabolic Diseases, Ruijin Hospital, Shanghai Jiao Tong University School of Medicine, No.197 Ruijin Second Road, Shanghai, 200025 China; 5 Key Laboratory for Endocrine and Metabolic Diseases of the National Health Commission of the PR China, Shanghai National Clinical Research Center for Metabolic Diseases, Shanghai National Center for Translational Medicine, Ruijin Hospital, Shanghai Jiao Tong University School of Medicine, No.197 Ruijin Second Road, Shanghai, 200025 China

**Keywords:** circ_072697, Diabetic foot ulcer, Keratinocytes, miR-3150a-3p, KDM2A, MAPK signaling pathway

## Abstract

**Objective:**

Diabetes foot ulcer (DFU) is a serious complication of diabetes, which can lead to significant mortality and amputation rate. Our previous study found circ_072697 was highly expressed in DFU tissues, but the regulatory mechanism of circ_072697 in DFU remains unclear.

**Methods:**

The relative expressions of circ_072697, miR-3150a-3p, and KDM2A in DFU patients or advanced glycation end products (AGEs)-treated HaCaT cells (used as DFU cell model) were determined by using qRT-PCR. Cell proliferation and migration abilities were determined by using CCK-8 and Transwell assays. The interaction between miR-3150a-3p with circ_072697 or KDM2A were verified by RNA immunoprecipitation (RIP) and dual-luciferase reporter assays. Furthermore, the protein expression of genes involved in MAPK signaling pathway was detected by western blot.

**Results:**

The expression of circ_072697 was significantly upregulated in DFU tissues, while the expression of miR-3150a-3p was downregulated. Circ_072697 knockdown promoted the proliferation and migration of AGEs-treated HaCaT cells. miR-3150a-3p was confirmed as a target of circ_072697 and its inhibitor reversed the promotion effects of circ_072697 knockdown on biological behavior of cells. In addition, KDM2A was considered as a target of miR-3150a-3p and it was highly expressed in DFU samples. Importantly, circ_072697 could regulate KDM2A expression through sponging miR-3150a-3p, and this axis had effect on the MAPK signaling pathway.

**Conclusions:**

Overall, circ_072697 regulated the biological behaviors of keratinocytes in DFU via miR-3150a-3p/KDM2A axis and MAPK signaling pathway, revealing a new insight into the pathogenesis and potential therapeutic targets of DFU.

**Supplementary Information:**

The online version contains supplementary material available at 10.1186/s12902-023-01430-2.

## Introduction

Diabetic foot ulcer (DFU) is a prevalent complication of diabetes, which is an important factor leading to disability and death of diabetes patients. It is reported that 19–34% of diabetes may be affected by DFU in their lifetime [1]. Patients with DFUs have a lower quality of life and poorer psychological adjustment, which imposes a huge economic burden on families and society [2]. Currently, the treatment of DFU follows the traditional protocol, including reducing foot pressure, protecting ulcer wound, restoring tissue perfusion, preventing infection, and controlling blood glucose, etc. [3, 4]. Although some positive effects have been achieved, the ulcer healing rate is still unsatisfactory, so there is an urgent clinical need to find new treatment strategies to improve the healing rate of DFU. In recent years, novel biotherapies have shown an excellent application prospect in the wound treatment of DFU and become an effective way to improve the healing rate.

As a noncoding RNA, circRNAs are abundant in human body and serve an important role in the occurrence and processes of various diseases. Due to their special circular structure, circRNAs are more stable and resistant to degradation than mRNA [5]. With the development and application of bioinformatics technology, the important role of circRNAs in the organism have been confirmed, and abnormal expression of several circRNAs has been reported to be closely linked to the disease process of diabetes. For example, circ_0000064 can enhance the fibrosis and proliferation of mesangial cells in diabetic nephropathy tethered cells [6], circ_001209 can aggravate diabetic retinal vascular dysfunction [7], and circ_0084443 can regulate the migration and proliferation of keratinocytes in DFU [8]. Thus, the regulation of circRNA may be a key mechanism for future DFU therapy.

MiRNAs can be regulated by circRNA to participate in organism metabolism, which is of great significance to biological processes such as gene expression and transcription. circRNAs function as the sponge of miRNAs to play a role of competitive endogenous RNA (ceRNA) to regulate gene expression. A recent study reported by Zeng et al. [9] has explored several ceRNAs related to angiogenesis and infectious inflammation of DFU, such as circ-0049271/miR-24–3p/JUNB. Other studies have also screened new diagnostic biomarkers from ceRNA regulatory network of DFU [10, 11]. These researches highlight the important role of circRNA/miRNA/mRNA regulatory mechanisms in the DFU pathogenesis. Interestingly, our previous bioinformatics analyses found that circ_072697 acted as miR-3150a-3p sponge to accelerate the progress of DFU through regulating mitogen-activated protein kinase (MAPK) signaling pathway [12]. Meanwhile, our previous study observed that lysine demethylase 2 A (KDM2A) might be a potential target of miR-3150a-3p [12]. It is reported that KDM2A plays an important role in chromosome remodeling and gene transcription, which is involved in the development and maintenance of keratinocytes proliferation [13, 14]. Keratinocytes have active processes during wound healing and angiogenesis, so abnormal expression of genes participating in keratinocytes migration and proliferation may contribute to impaired wound healing in diabetes [15]. However, the explicit function of circ_072697/miR-3150a-3p/KDM2A axis in DFU is still imprecise.

In this research, we investigated the expression of circ_072697 in DFU tissues as well as cells, and then performed in vitro functional experiments to explore the potential mechanisms of circ_072697 in regulating keratinocytes growth and MAPK signaling pathway in DFU pathogenesis. Results showed that circ_072697 promoted the occurrence of DFU by sponging miR-3150a-3p and targeting KDM2A. Taken together, this study contributed to our further understanding of the biological function of circ_072697 in keratinocytes proliferation and may serve as a novel target for DFU treatment.

## Materials and methods

### Participants and clinical sample collection

Foot wound tissues from eight DFU patients (DFU group) and eight non-diabetic patients (normal group) were recruited from Ruijin Hospital, Shanghai Jiaotong University School of Medicine. Inclusion criteria for DFU patients were as follows: patients with the age over 18 years old; patients diagnosed as diabetes according to the 1999 World Health Organization criteria for diabetes mellitus and the duration of diabetes was more than 10 years [16]; foot ulcers meeting Wagner Classification Standard Grade II-III [17]; patients with hemoglobin A1c (HbA1c) ≥ 7.5%. Patients were excluded if they had pacemaker, febrile disease, and drug allergy history. The wound samples from these subjects were collected. All processes were performed based on the Declaration of Helsinki [18] and received ethics approval from ethics committee of Ruijin Hospital, Shanghai Jiaotong University School of Medicine (2019-85). The written informed consents were obtained from participants prior to the start of this research.

### Cell culture and treatment

Advanced glycation end products (AGE)-induced keratinocytes were applied to construct the in vitro DFU model. HaCaT cells (Human keratinocyte cells) were purchased from Shanghai Sunteam Biotechnology Co., Ltd (CAT: sunH8751; Shanghai, China) and routinely cultured in Dulbecco’s Modified Eagle’s medium (DMEM, BasalMedia, Shanghai, China) added with 10% fetal bovine serum (FBS, BasalMedia) and 1% Penicillin-Streptomycin (BasalMedia) at 37 °C cell incubator with 5% CO_2_. When the cells grew to approximately 30–40% confluence, they were treated with 60 mg/mL AGE-HSA, and then, cells in log phase were taken for further analysis.

### SiRNAs and cell transfection

Three small interfering RNAs targeting circ_072697 (si-circ #1: ATAACCAGCCACTTCATATTT, si-circ #2: GACAGTGTGAGTGGATCTATT, and si-circ #3: ACATGCTGAAACTTGGCTATT), negative control for siRNA (si-NC: UUCUCCGAACGUGUCACGUTT), miR-3150a-3p inhibitor (miR-3150a-3p inh), and negative control for miR-3150a-3p inhibitor (inh-NC) were transfected into AGE-treated HaCaT cell, according to the manufacturer’s instructions. Lipofectamine 2000 (Invitrogen) was employed to conduct cell transfection. After 48 h reaction, cells were collected for RNA extraction to verify the transfection efficiency.

### Cell counting kit-8 (CCK-8) assay

The cells samples were transferred to a 96-well cell plate and cultured for 24 h, 48 and 72 h, respectively. After discarding old medium, 100 µL of fresh medium with 10 µL of CCK-8 reagent was added to each well for further 2 h incubation. Absorbance at 450 nm was recorded under the microplate reader.

### Transwell assay

The 24-well Transwell chamber was employed to conduct cell migration assay. In brief, 200 µL cell suspension and 600 µL culture medium (containing 10% FBS) was added into the upper and lower chamber, respectively. After 12 h incubation at 37 °C cell incubator (5% CO_2_), the non-invaded cells were carefully wiped off. Invaded cells under the membrane were fixed with 4% paraformaldehyde for 20 min and then stained with crystal violet solution for 15 min. The inverted microscope was used for photographing and observing, and the number of invaded cells was also counted.

### Real-time PCR (RT-qPCR) assay

Total RNA from DFU samples and cells was extracted using Trizol reagent (Thermo Fisher Scientific). The purity and concentration of the extracted RNA were determined using a NanoDrop 2000c spectrophotometer (Thermo Scientific). Then, RNA was reverse transcribed into complementary DNA (cDNA) using a PrimeScript™ RT reagent Kit with gDNA Eraser (Takara Bio, Dalian, China). Next, RT-qPCR was performed by SYBR® Premix Ex Taq™ reagent (Takara Bio, Dalian, China) with the ViiA™7 Real-Time PCR System (Thermo Fisher Scientific). GADPH as internal reference for mRNA and circ_072697 detection, while U6 as reference for miR-3150a-3p. Table 1 lists the primer sequences.

**Table 1:** Primer sequences of RT-qPCR

Gene name	Primer sequence
Circ_072697	Forward: 5’-GTATGAGCGCAGTTATGCATAGA-3’
	Reverse: 5’-TCCCAGGTGACTACGAAAATGTT-3’
miR-3150a-3p	Forward: 5’-CGCTGGGGAGATCCTCGA-3’
	Reverse: 5’-AGTGCAGGGTCCGAGGTATT-3’
KDM2A	Forward: 5’-CCGATTGTGTCAGGAGCCAG-3’
	Reverse: 5’-CACAGGACTGCTTCATGCGTC-3’
U6	Forward: 5’-CTCGCTTCGGCAGCACAT-3’
	Reverse: 5’-CTCGCTTCGGCAGCACAT-3’
GAPDH	Forward: 5’-GTCTCCTCTGACTTCAACAGCG-3’
	Reverse: 5’-ACCACCCTGTTGCTGTAGCCAA-3’

### Western blot assay

Total proteins were extracted from tissues or cells with radioimmunoprecipitation assay (RIPA) lysis buffer and added into sodium dodecyl sulfate‐polyacrylamide gel electrophoresis (SDS-PAGE) gels for electrophoresis. After being transferred to the polyvinylidene difluoride (PVDF) membrane, it was blocked with Tris buffered saline Tween (TBST) containing 5% non-fat milk for 1 h at room temperature and then reacted with the following primary antibody overnight at 4 °C: anti-KDM2A (Abcam, #ab191387), anti-ERK (#ab184699), anti-p-ERK (#ab201015), anti-p38 (#ab182453), anti-p-p38 (#ab178867), anti-JNK (#ab110724), anti-p-JNK (#ab215208), and GADPH (#ab8245). Subsequently, the PVDF membrane was washed with TBST and incubated with secondary antibody for 1 h. Proteins were identified using enhanced chemiluminescence (ECL) reagent and captured by ImageQuant LAS 4000 chemiluminescent imaging system (General Electric, MA, US). GADPH was applied as loading control. The bands were visualized by an enhanced chemiluminescence detection system (Tanon, Shanghai, China), and the gray value of each band was analyzed using ImageJ software (version 1.49; NIH, USA).

### RNA immunoprecipitation (RIP) assay

Magna RIP Kit (Millipore, MA, USA) was applied to conduct RIP assay with the guidance of the manufacturer’s instruction. In brief, transfected cells were washed with precooled PBS and lysed with RIP lysis solution. Then, cell lysates were incubated overnight at 4 °C with protein magnetic beads conjugated to the antibody. The purified immunoprecipitated RNA was detected using RT-qPCR.

### Dual luciferase reporter assay

Dual luciferase reporter assay was employed to assess the interaction between circ_072697 and miR-3150a-3p as well as KDM2A and miR-3150a-3p. The target gene was inserted into a Renilla luciferase gene vector, and then the vector was co-transfected cells with miRNA. After 48 h of transfection, cells were lysed with PLB lysis solution, and the relative luciferase value was determined.

### Characterization of circ_072697 in AGE-treated HaCaT cells

The cell nucleus and cytoplasm from AGE-treated HaCaT cell were separated and purified. In brief, weak ionic lysis solution was added to the cells, and the cells were blown 20–40 times with a pipette, vigorously shaken for 30 s, and bathed in ice for 15 min, followed by observation of cell rupture under a scientific microscope. The obtained lysis products were centrifuged at 1000 × g at 4 °C for 5 min, and the supernatant was collected as the cytoplasmic fraction. After adding 100 µl of weak lysate to the precipitated nuclear extract, the supernatant was discarded after centrifugation at 1000 × g for 5 min in an ice bath. Then, 100 µl of pre-cooled strong lysis solution was added to the precipitate and the supernatant was extracted after centrifugation at 12,000 × g for 5 min to obtain the nuclear fraction. The cytoplasmic and nucleus supernatants were subjected to RNA extraction and RT-qPCR validation, respectively. In addition, the circularized structure of circ_072697 was also confirmed using RT-qPCR.

### Statistical analysis

Statistical analysis was performed by using SPSS 23.0 software, and the data between two groups were compared by t-test. Data results were expressed as mean ± standard deviation, and *P* < 0.05 indicated the statistically significant.

## Results

### Characterization and expression pattern of circ_072697

**Fig. 1:**
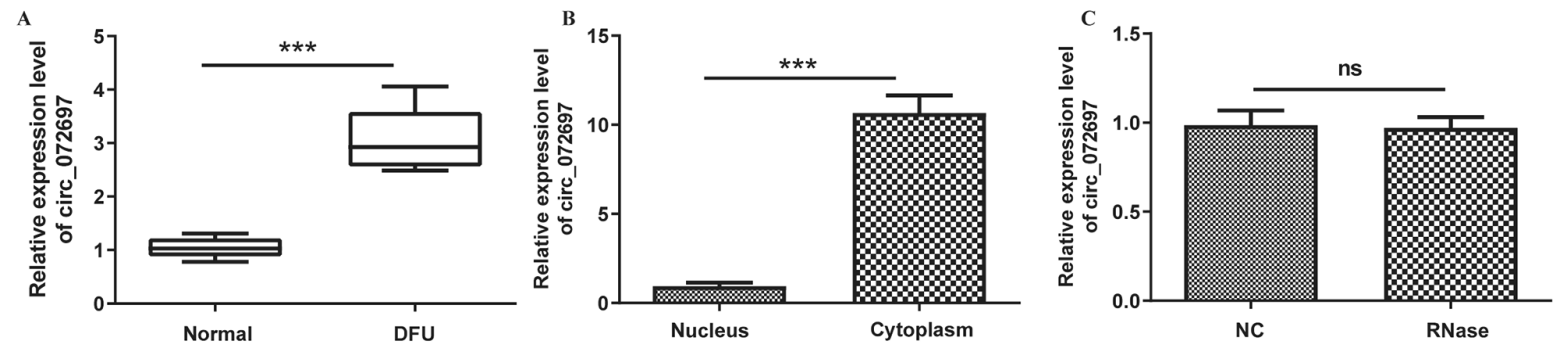
Identification and characteristics of circ_072697 in DFU samples. **A**: The expression level of circ_072697 were detected by qRT-PCR in DFU and normal samples (n = 8). **B**: Relative expression level of circ_072697 was measured by qRT-PCR in the nucleus and cytoplasm of AGE-treated HaCaT cells. C: Relative expression level of circ_072697 was detected by qRT-PCR in AGE-HaCaT cells after treatment of RNase R. Data are presented as the mean ± SD. *** p < 0.001; ns, not significant

Our previous bioinformatics analysis revealed that circ_072697 was observably upregulated in DFU tissues and may be served as a potential biomarker of DFU [12]. This study, we detected the expression level of circ_072697 in eight DFU patients and eight normal patients using RT-qPCR. Result verified that circ_072697 expression level was significantly elevated in DFU patients (p < 0.001, Fig. 1A). Next, the subcellular localization of circ_072697 was assessed. After separation of AGE-treated HaCaT cells into nuclear and cytoplasmic lysates, qRT-RCR analysis showed that circ_072697 was predominantly localized in the cytoplasm **(**Fig. 1B). Moreover, RNase R digestion assay indicated that RNase R + had little effect on the relative expression of circ_072697 (Fig. 1C). These data confirmed that circ_072697 was a circular RNA and stably located in the cytoplasm.

### Knockdown of circ_072697 promoted cell proliferation and migration of AGE-treated HaCaT cells

**Fig. 2:**
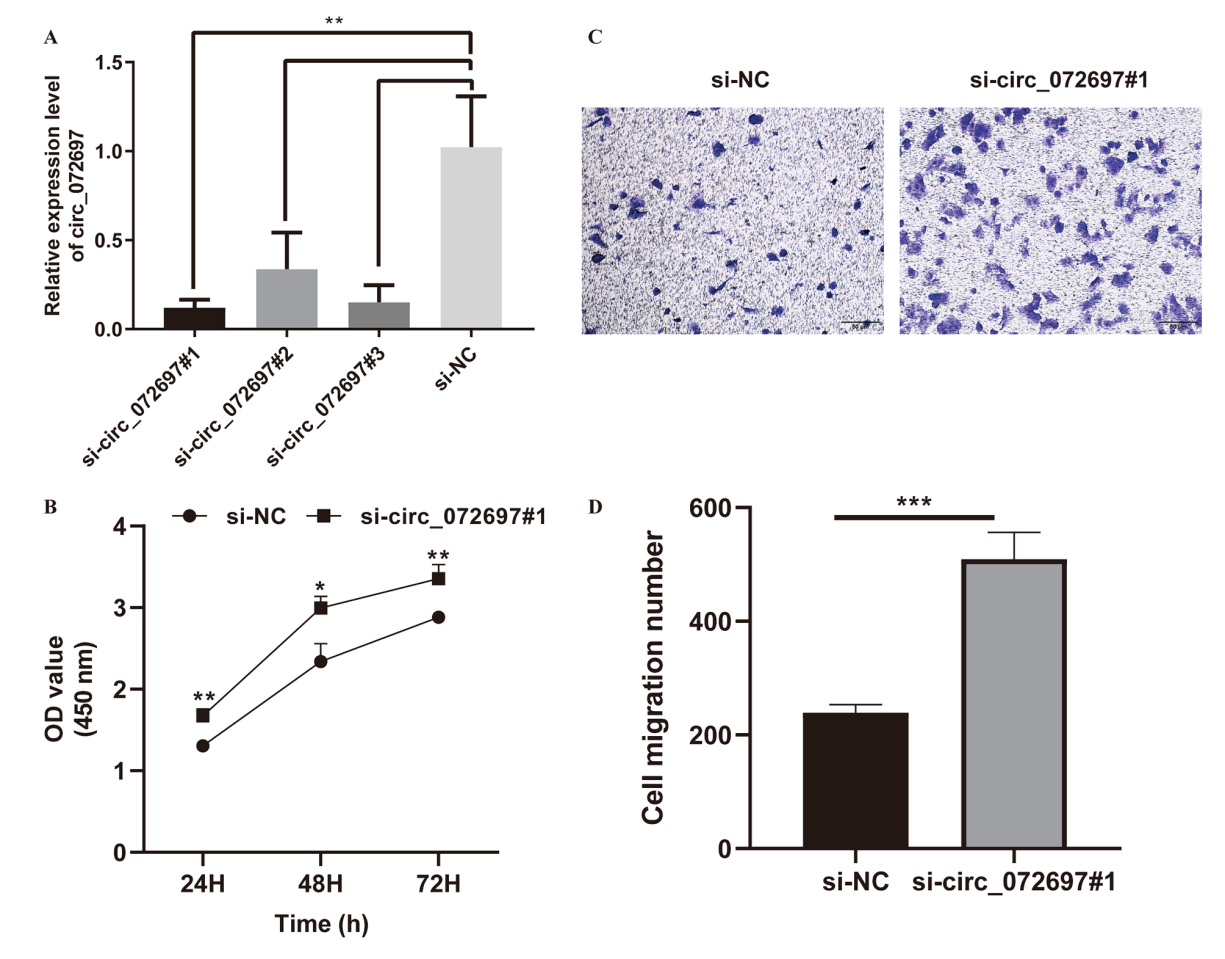
Knockdown of circ_072697 promoted proliferation and migration of AGE-treated HaCaT cells. **A**: Knockdown efficiency of circ_072697 (siRNA#1, siRNA#2, and siRNA#3) in HaCaT cells was detected using qRT-PCR. **B**: Cell viability was detected in cells after transfection for 24, 48, and 72 h using CCK-8 assay. **C**: Cell migration were tested using Transwell assay (scale bar, 50 μm). **D**: Counting of cell migration number. * p < 0.05, ** p < 0.01, *** p < 0.001

To explore the biological functions of circ_072697 in DFU, the circ_072697 was knocked down via transferring three circ_072697 siRNAs (siRNA#1, siRNA#2, and siRNA#3) into AGE-treated HaCaT cells. Results showed that the expression level of circ_072697 was distinctly decreased in AGE-treated HaCaT cells after siRNA treatment, especially in siRNA#1 (p < 0.01, Fig. 2A). Hence, siRNA#1 was selected for further research. Next, CCK-8 assay revealed that knockdown of circ_072697 could significantly enhanced cell proliferation (p < 0.05, Fig. 2B). Meanwhile, transwell assay showed that knockdown of circ_072697 significantly increased the number of migrating cells (p < 0.001, Fig. 2C and D). Collectively, the above data showed that circ_072697 knockdown could promote cell proliferation and migration of AGE-treated HaCaT cells.

### circ_072697 served as a sponge of miR-3150a-3p in AGE-treated HaCaT cells

**Fig. 3:**
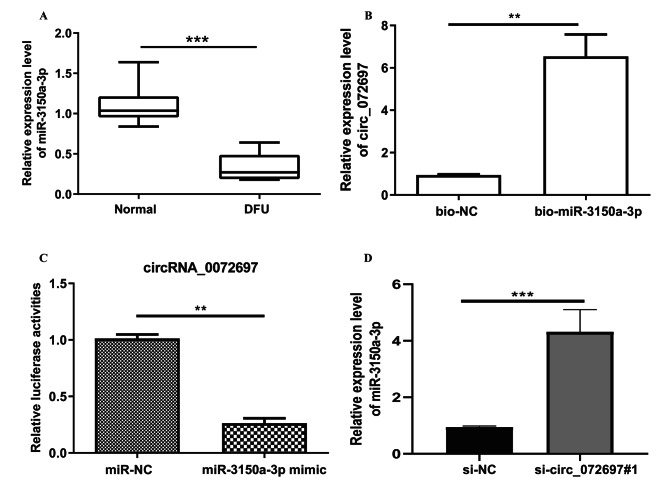
circ_072697 directly targeted miR-3150a-3p in AGE-treated HaCaT cells. **A**: The expression level of miR-3150a-3p in HaCaT cells was detected using qRT-PCR. **B**: RIP assay was employed to assess the interaction of circ_072697 and miR-3150a-3p. **C**: The luciferase activities in HaCaT cells co-transfected with miR-3150a-3p NC or miR-3150a-3p mimics. **D**: The miR-3150a-3p level was detected in cells with si-NC or si-circ_072697#1. ** p < 0.01, *** p < 0.001

Our previous study predicted target miRNAs of circ_072697 and found that miR-3150a-3p had complementary binding sites with circ_072697 [12]. In this research, miR-3150a-3p exhibited significantly lower expression in the DFU tissues than those in the normal tissues (p < 0.001, Fig. 3A). To further confirm the correlation between circ_072697 and miR-3150a-3p, the RIP and dualluciferase reporter assays were conducted. RIP assay indicated that miR-3150a-3p could directly bind to circ_072697 (Fig. 3B). Luciferase experiment revealed that the luciferase intensity of circ_072697 and miR-3150a-3p co-transfection group was significantly decreased than that of the control group (p < 0.01, Fig. 3C). In addition, the expression level of miR-3150a-3p in cells was significantly elevated after knockdown of circ_072697 (p < 0.001, Fig. 3D). Altogether, the above results revealed that circ_072697 served as a miR-3150a-3p sponge.

### Inhibition of miR-3150a-3p reversed the effect of circ_072697 knockdown on AGE-treated HaCaT cells

**Fig. 4:**
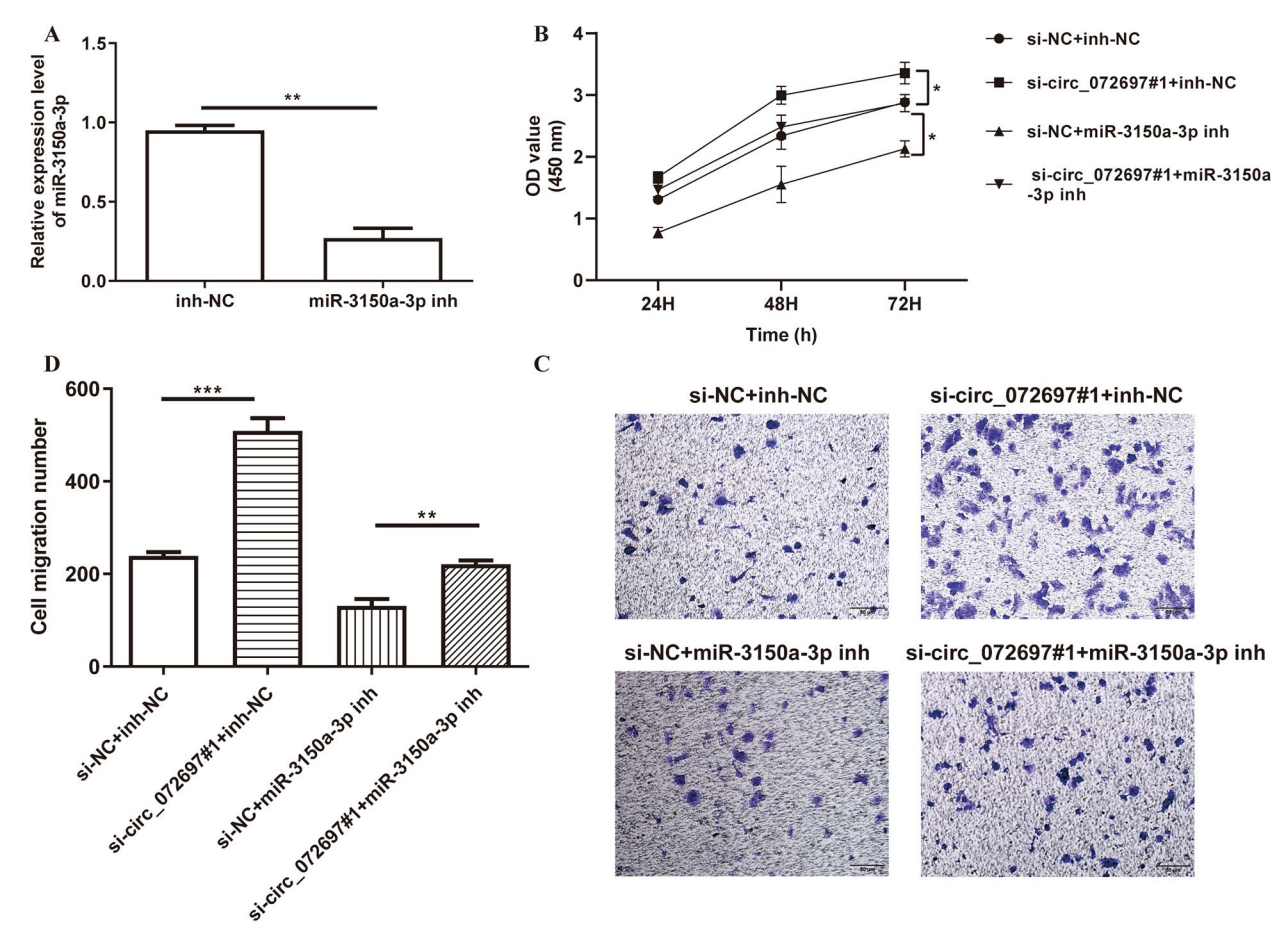
circ_072697 regulated AGE-treated HaCaT cell proliferation and migration by directly targeting miR-3150a-3p. **A**: The relative expression level of miR-3150a-3p in cells transfected with inh-NC or miR-3150a-3p inhibitor. **B**: CCK-8 assay showed the OD value of cells transfected with si-NC + inh-NC, si-circ_072697#1 + inh-NC, si-NC + miR-3150a-3p inh, or si-circ_072697#1 + miR-3150a-3p inh in 24 h, 48 and 72 h. **C**: Transwell assay showed the cell migration ability. **D**: Determination of cellular migration number in different groups. * p < 0.05, ** p < 0.01, *** p < 0.001

To explored the biological role of miR-3150a-3p in DFU pathogenesis, we established stably miR-3150a-3p-silencing AGE-treated HaCaT cells. The expression level of miR-3150a-3p was verified by qRT-RCR, and results showed that miR-3150a-3p expression was significantly decreased in cells transfected with miR-3150a-3p inhibitor (p < 0.01, Fig. 4A). Next, AGE-treated HaCaT cells were transfected with si-circ_072697#1 and/or miR-3150a-3p inhibitor. We observed that the acceerative effects of si-circ_072697 on cell viability (Fig. 4B) and cell migration (Fig. 4C and D) in AGE-treated HaCaT cells were all reversed by miR-3150a-3p inhibitor. Altogether, these findings indicated that circ_072697 promoted cell proliferation and migration by interacting with miR-3150a-3p.

### Circ_072697 could regulate the KDM2A expression via miR-3150a-3p

**Fig. 5:**
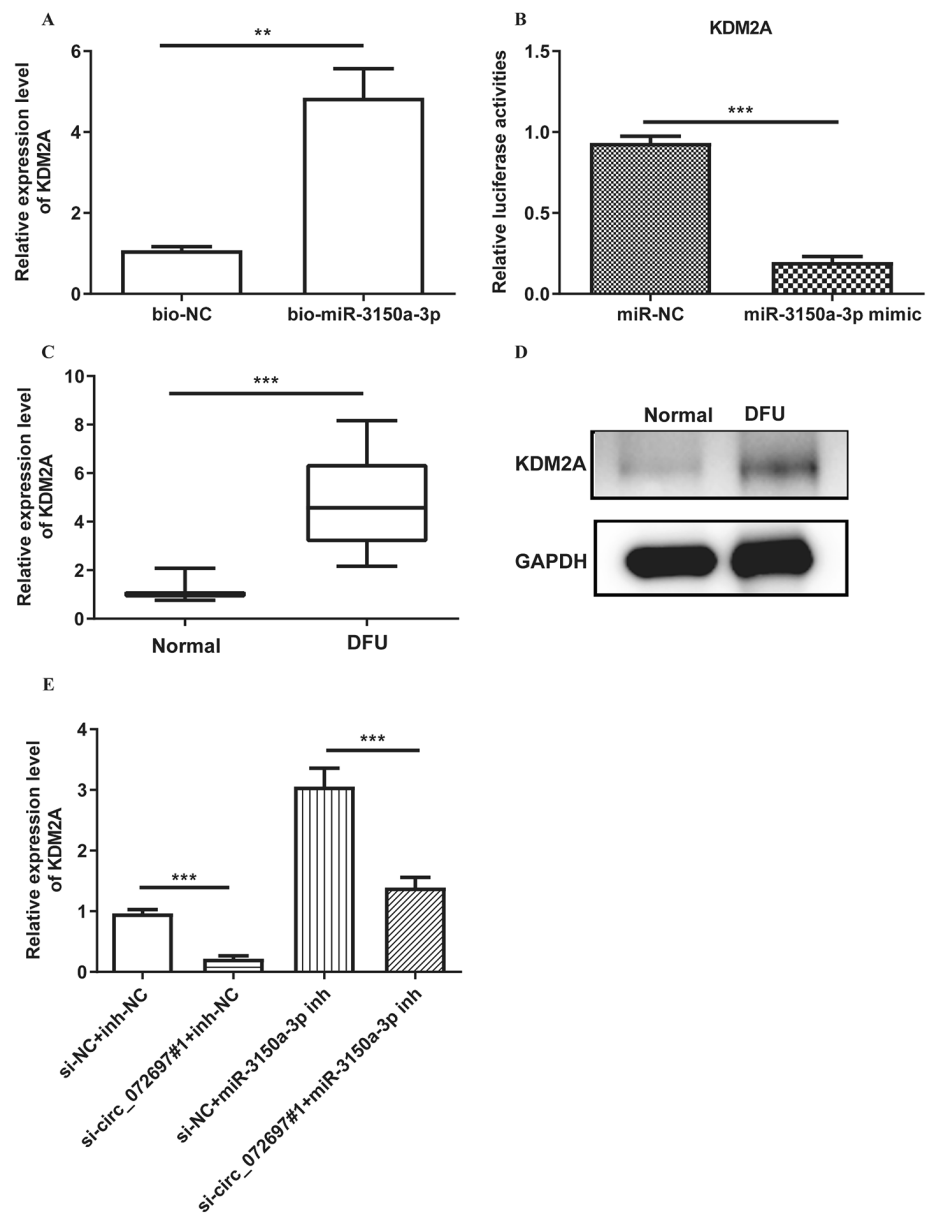
Circ_072697 regulated KDM2A expression by sponging miR-3150a-3p. **A**: RIP assay was employed to assess the interaction of KDM2A and miR-3150a-3p. **B**: The luciferase activities in HaCaT cells co-transfected with miR-3150a-3p NC or miR-3150a-3p mimics. **C**: qRT-PCR of KDM2A expression in normal and DFU tissues. **D**: Western blot analysis of KDM2A expression in normal and DFU tissues. **E**: qRT-PCR analysis of KDM2A expression in AGE-treated HaCaT cells transfected with si-NC + inh-NC, si-circ_072697#1 + inh-NC, si-NC + miR-3150a-3p inh, or si-circ_072697#1 + miR-3150a-3p inh. ** p < 0.01, *** p < 0.001

Our previous study also indicated that KDM2A as a potential target of miR-3150a-3p [12]. To determine whether miR-3150a-3p could bind to the KDM2A, RIP assay and dual-luciferase reporter assay were conducted. RIP assay showed that the mRNA expression level of KDM2A was significantly increased in the Bio-miR-3150a-3p group (p < 0.01, Fig. 5A); dual luciferase reporter revealed that miR-3150a-3p mimic could inhibit the luciferase activity of KDM2A (p < 0.001, Fig. 5B), confirming an interaction between KDM2A and miR-3150a-3p. Further, KDM2A were significantly upregulated in the DFU tissues at the mRNA and protein levels (Fig. 5C and D). Besides, we explored whether circ_072697 served its biological function via circ_072697/miR-3150a-3p/KDM2A axis in AGE-treated HaCaTs cells. Results showed that circ_072697 knockdown increased the expression level of KDM2A, while this increase could be suppressed by miR-3150a-3p inhibitor (Fig. 5E). Taken together, circ_072697 could regulate KDM2A expression via binding with miR-3150a-3p.

### Circ_072697/miR-3150a-3p/KDM2A axis had effect on MAPK signaling pathway

**Fig. 6:**
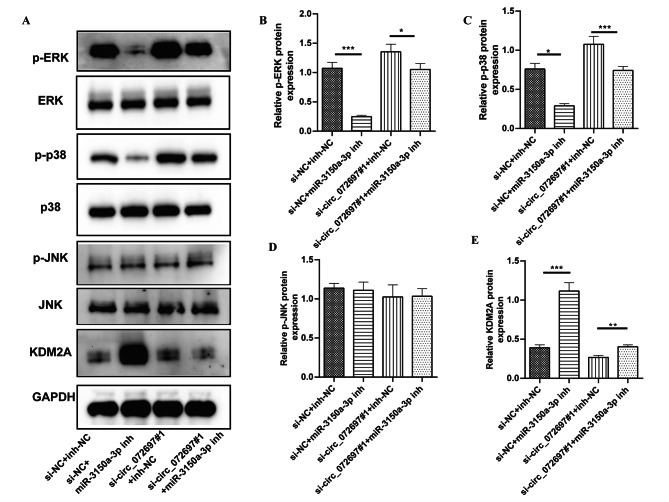
Circ_072697 knockdown promoted the cell proliferation via miR-3150a-3p/KDM2A axis by regulating MAPK signaling pathway. **A**: p-ERK, ERK, p-p38, p38, p-JNK, JNK, and KDM2A protein level detected by western blot. **B**: The relative protein level of p-ERK. **C**: The relative protein level of p-p38. **D**: The relative protein level of p-JNK. E: The relative protein level of KDM2A. * p < 0.05, ** p < 0.01, *** p < 0.001

**Fig. 7:**
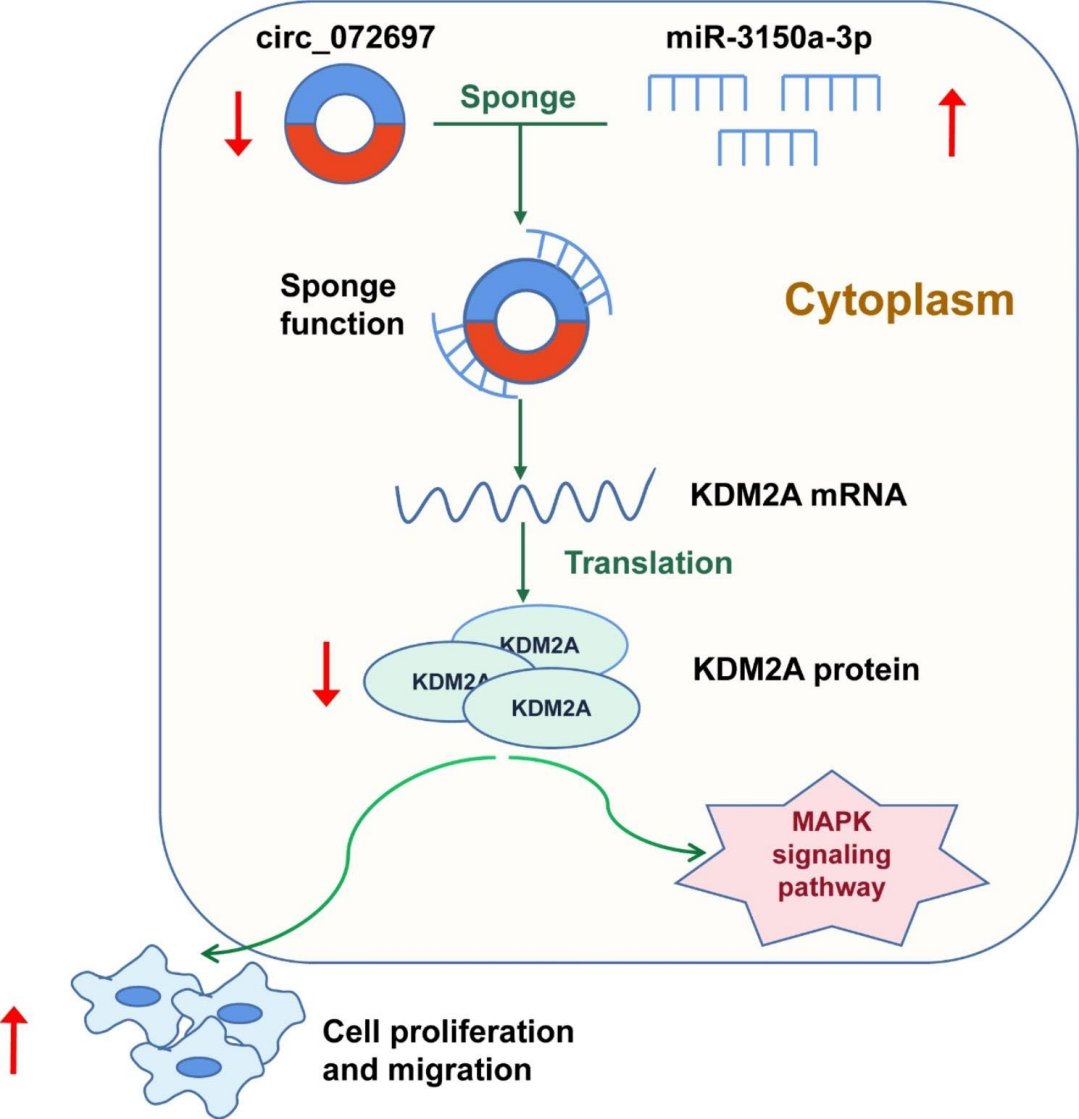
Schematic diagram of the regulatory mechanism of the circ_072697/miR-3150a-3p/KDM2A axis in mediating AGE-treated HaCaT cells proliferation and migration. Results showed that circ_072697 knockdown promoted HaCaT cells proliferation and migration through directly upregulating miR-3150a-3p and inhibiting KDM2A expression

Our previous study indicated that miR-3150a-3p was involved in the MAPK signaling pathway, which was contributed to DFU development [12]. Thus, we decided to verify whether circ_072697 could regulate the MAPK signaling pathway via miR-3150a-3p/KDM2A axis in AGE-treated HaCaTs cells. Western blot analysis showed that p-ERK and p-p38 protein levels were significantly increased after circ_072697 knockdown (p < 0.05), but this effect was abolished by miR-3150a-3p inhibitor (p < 0.05, Fig. 6A C). However, there was no significant difference in p-JNK protein level among these groups (Fig. 6D). Further, miR-3150a-3p inhibitor restored the circ_072697 knockdown-induced decrease in KDM2A (p < 0.05, Fig. 6E). Overall, circ_072697 regulated miR-3150a-3p/KDM2A axis to affect the MAPK signaling pathway in AGE-treated HaCaTs cells (Fig. 7).

## Discussion

DFU wound healing is influenced by multiple factors, and its mechanisms mainly involve hyperglycemic-induced angiogenesis disorders, abnormal inflammatory response, and changes in the migration and proliferation of fibroblasts and keratin-forming cells. The pathological process of DFU is extremely complex and difficult to therapy [19]. Conventional treatments, such as glycemic control and interventional surgery, have certain limitations [20]. Scientists have been working to find better treatments. Recent study revealed that the neutrophil-to-lymphocyte ratio could be used as a non-invasive biomarker to determine DFU development, highlighting the potential of biomarkers in improving the early diagnosis and management of DFU [21]. However, studies to screen for DFU-related biomarkers are still limited. Our previous studies have shown that circ_072697 is involved in the pathogenesis of DFU, but its biological function remains largely unknown [12]. In this study, qRT-PCR results showed that circ_072697 was stably upregulated in DFU tissues. In vitro experiments indicated that knockdown of circ_072697 significantly promoted the proliferation and migration of AGE-treated HaCaTs cells. RIP assay and dual luciferase reporter assays revealed that circ_072697 could directly bind to miR-3150a-3p. Moreover, we found that miR-3150a-3p was downregulated in DFU tissues, and KDM2A as a direct target of miR-3150a-3p. Western blot and qRT-PCR showed that KDM2A was upregulated in DFU tissues. Further study confirmed that knockdown of circ_072697 promoted cell proliferation and migration via regulating miR-3150a-3p/KDM2A. This regulatory relationship of ceRNA was first identified in DFU, and these findings suggested that circ_072697 might be a novel diagnostic and therapeutic target for DFU.

CircRNAs are important regulator factor in the cellular life cycle, playing critical roles in biological processes such as cell proliferation, apoptosis, and inflammation [22]. With the rapid development of circRNA field, the specific circRNAs in the wound tissues or blood of DFU patients have been detected by RNA sequencing. For example, our previous microarray analysis identified 65 differentially expressed circRNAs between non-diabetic and DFU patients, and Liao et al. screened eight differential circRNAs [10, 12]. These circRNAs may be involved in the DFU healing process and signaling pathways by regulating certain target genes. Notably, re-epithelialization is the process of wound healing and restoration of intact epidermis, that is closely regulated by migration and proliferation of keratinocytes [23]. Several circRNAs such as circ_0084443 have been confirmed to be significantly overexpressed in DFU patients and their knockdown increase keratinocytes migration [8, 24]. These studies highlight the importance of circRNAs in the exploration of etiological mechanism or treatment of DFU. In this study, we confirmed that circ_072697 was a circular RNA and stably localized in the cytoplasm. Interestingly, circ_072697 silencing promoted the proliferation and migration of HaCaT cells by downregulating KDM2A expression via sponging miR-3150a-3p. Evidence has indicated that HaCaT cells (human keratinocytes) are constantly renewed in the epidermis through differentiation, proliferation, and migration, thereby facilitating wound healing [25]. Importantly, promotion of local keratinocyte proliferation and wound migration may improve the healing disorder of DFU to some extent. Based on these researches, we speculate that circ_072697 may be a potential therapeutic target for a DFU. However, whether it enhances wound healing needs to be validate in clinical samples.

KDM2A gene encodes a member of the F-box protein family, and its aberrant expression can be observed in a variety of cancers, such as gastric cancer [26]. Tanka et al. [27] described a potential correlation between KDM2A and diabetes. They found that metformin (a drug used to treat type 2 diabetes) activated KDM2A by increasing AMPK activity, which led to a decrease in cell proliferation. However, its contribution to the pathogenesis of DFU has not been reported. For some non-healing DFU, the main features are low-grade chronic inflammation and proliferation that fails to progress to wound healing [28]. KDM2A has been confirmed to regulate the inflammatory reaction of keratinocytes in psoriasis [29]. Thus, we speculate that KDM2A may exacerbate the symptoms of DFU by increasing the inflammatory response. However, the specific molecular mechanism needs to be further explored.

Further, circ_072697 knockdown activated several genes involved in MAPK signaling pathway, such as p-ERK, p-p38. MAPK signaling pathway can promote the healing of damaged skin tissue by activating ERK and p38 signals to enhance intercellular information transmission [30]. It had been proved that activated ERK promoted the proliferation of wound fibroblasts, vascular endothelial cells and epithelial cells by regulating downstream signals c-myc and Akt, while activated p38 could participate in angiogenesis by improving vascular permeability and enhancing the proliferation and migration of vascular endothelial cells [31]. In this study, the MAPK pathway was activated after circ_072697 knockdown, and the protein expressions of p-ERK and p-P38 were significantly increased. The results reflected that MAPK signaling pathway might be inhibited during the development of DFU.

In short, we had determined that circ_072697 was highly expressed in DFU chronic nonhealing wounds, and formed a circ_072697 - miR-3150a-3p - KDM2A ceRNA regulatory network. The results strongly support the function and potential clinical significance of circ_072697 in skin wound healing. Further efforts are warranted to explore whether circ_072697 exerts biological effects in DFU through other mechanisms, which will contribute to our new understanding of DFU pathophysiology.

## Conclusions

In conclusion, we observed that circ_072697 knockdown facilitated the proliferation and migration of keratinocytes through miR-3150a-3p/KDM2A axis. These findings indicated that circ_072697 might be a potential biomarker for the diagnosis or treatment of DFU.

### Electronic supplementary material

Below is the link to the electronic supplementary material.

Supplementary Material 1

## Data Availability

All data generated or analysed during this study are included in this published article and its supplementary information files.

## Abbreviations

DFU: Diabetes foot ulcer
KDM2A: Lysine Demethylase 2 A
MAPK: Mitogen-activated protein kinase
AGEs: Advanced glycation end products
RIP: RNA immunoprecipitation
ceRNA: Competitive endogenous RNA
HbA1c: Hemoglobin A1c
DMEM: Dulbecco’s Modified Eagle’s medium
FBS: Fetal bovine serum
CCK-8: Cell counting kit-8
RT-qPCR: Real-time PCR
cDNA: Complementary DNA
RIPA: Radioimmunoprecipitation assay
SDS-PAGE: Sodium dodecyl sulfate-polyacrylamide gel electrophoresis
PVDF: Polyvinylidene difluoride
TBST: Tris buffered saline Tween
ECL: Enhanced chemiluminescence
RIP: RNA immunoprecipitation
