# Supplementary material for: Circ_072697 knockdown promotes advanced glycation end products-induced cell proliferation and migration in HaCaT cells via miR-3150a-3p/KDM2A axis
Source: BMC Endocr Disord. 2023 Sep 19;23:200. doi: 10.1186/s12902-023-01430-2 (PMC10507952; doi:10.1186/s12902-023-01430-2)
Supplement: Supplementary file 1 — Supplementary Material 1 [file 12902_2023_1430_MOESM1_ESM.pdf]

The original WB bands of Figure 5

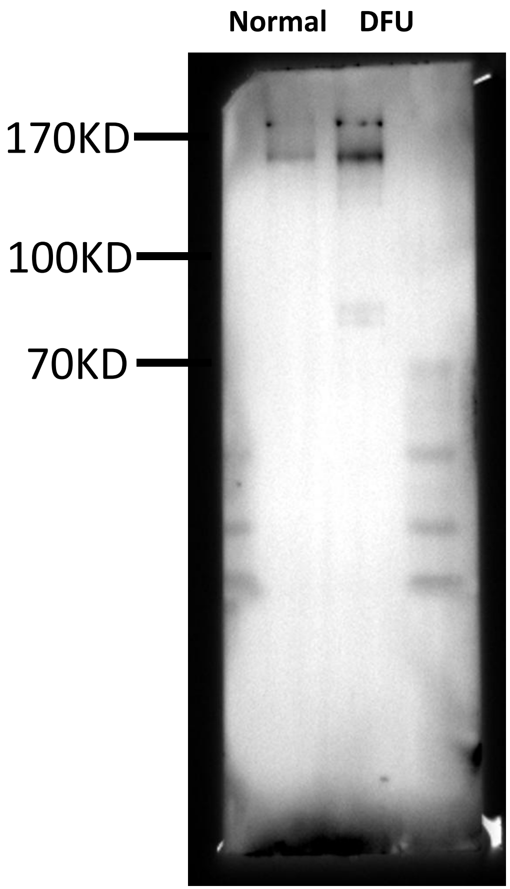

KDM2A

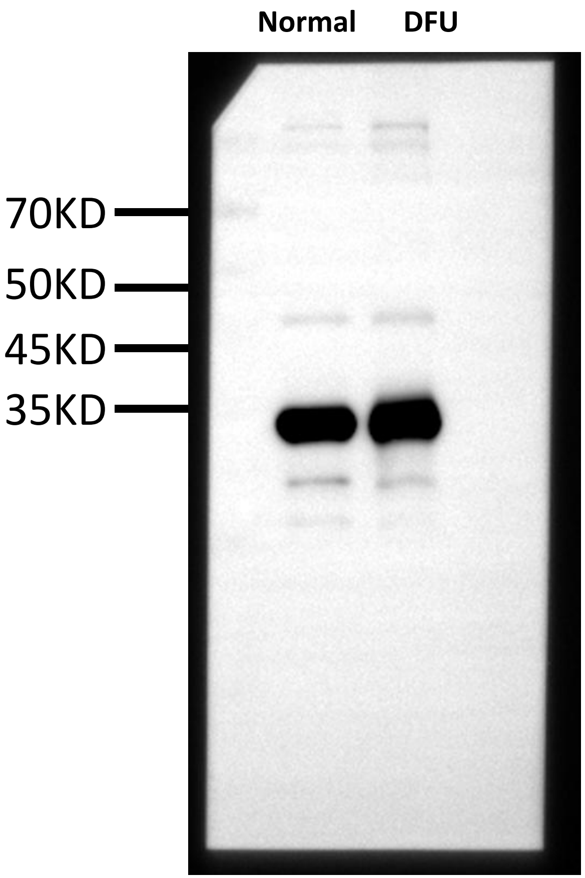

GAPDH

The original WB bands of Figure 6

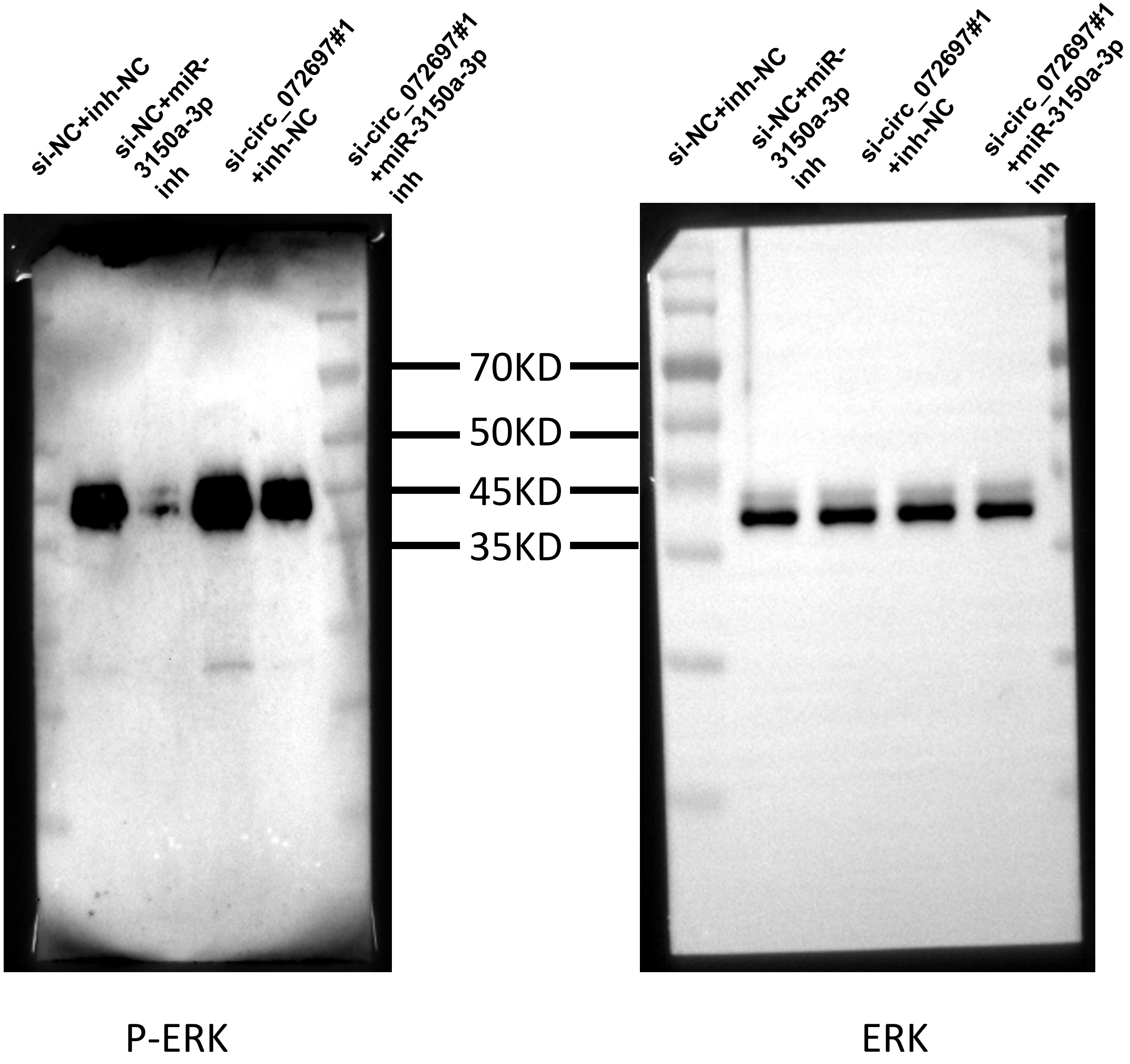

The original WB bands of Figure 6

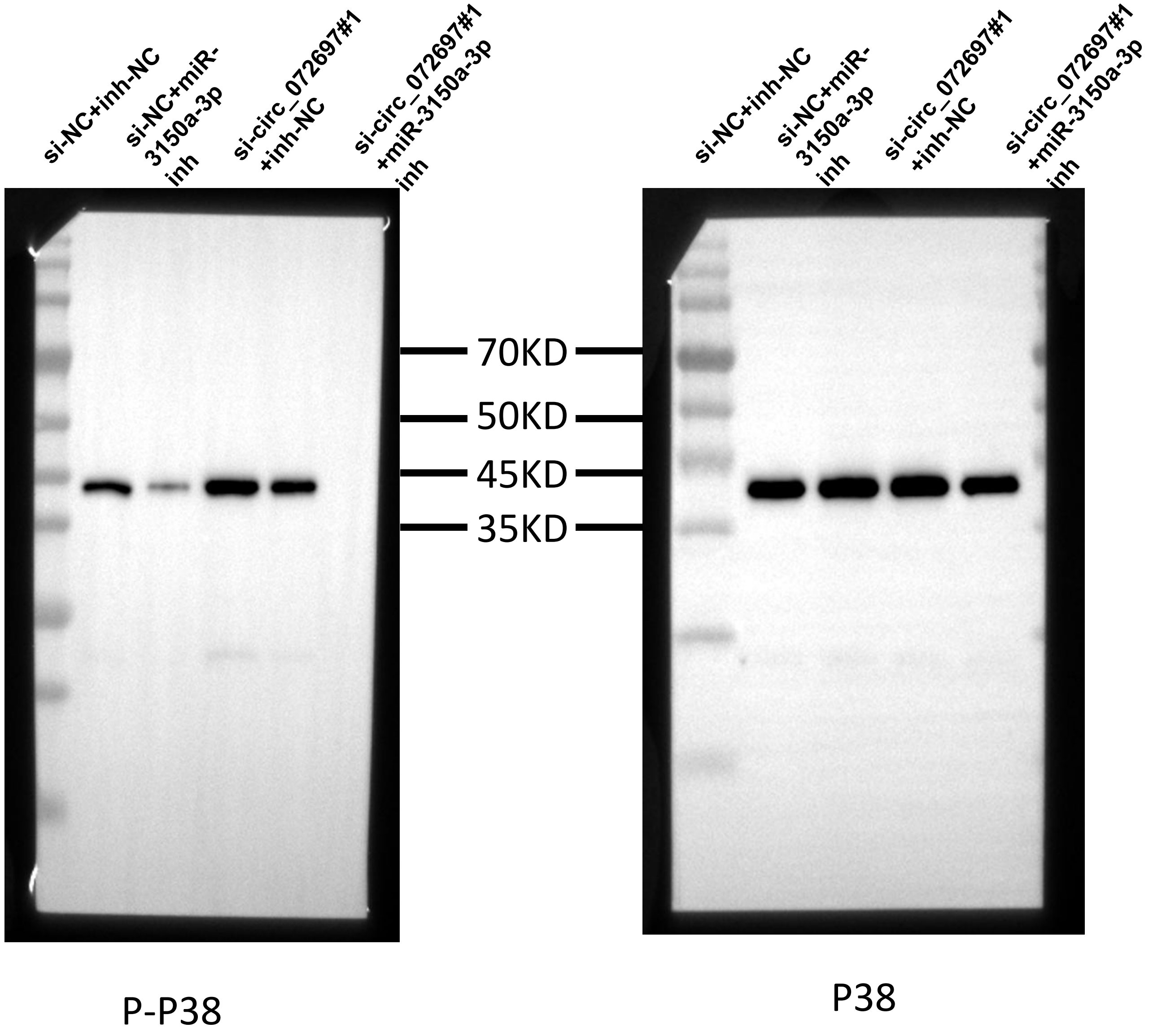

The original WB bands of Figure 6

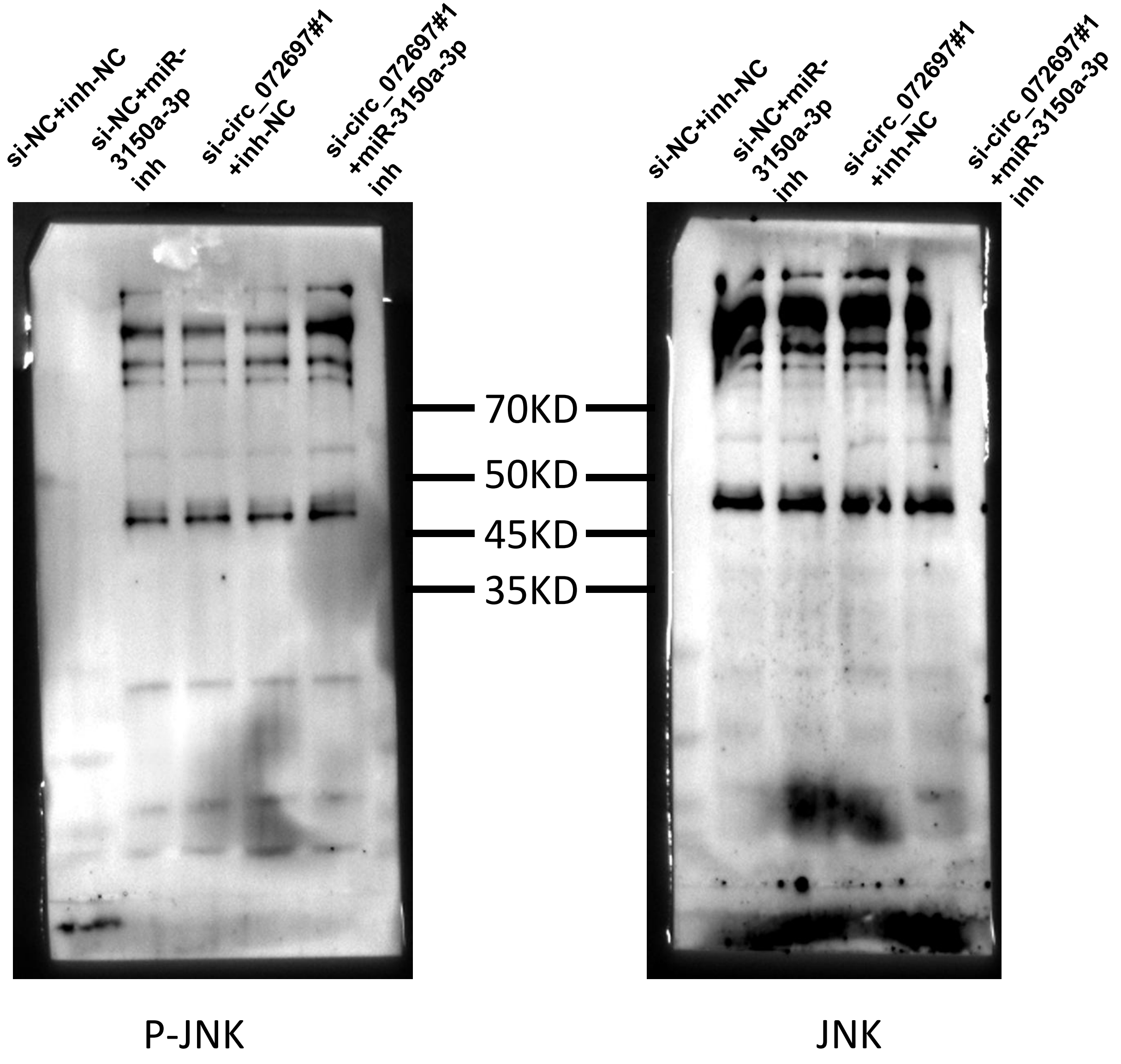

The original WB bands of Figure 6

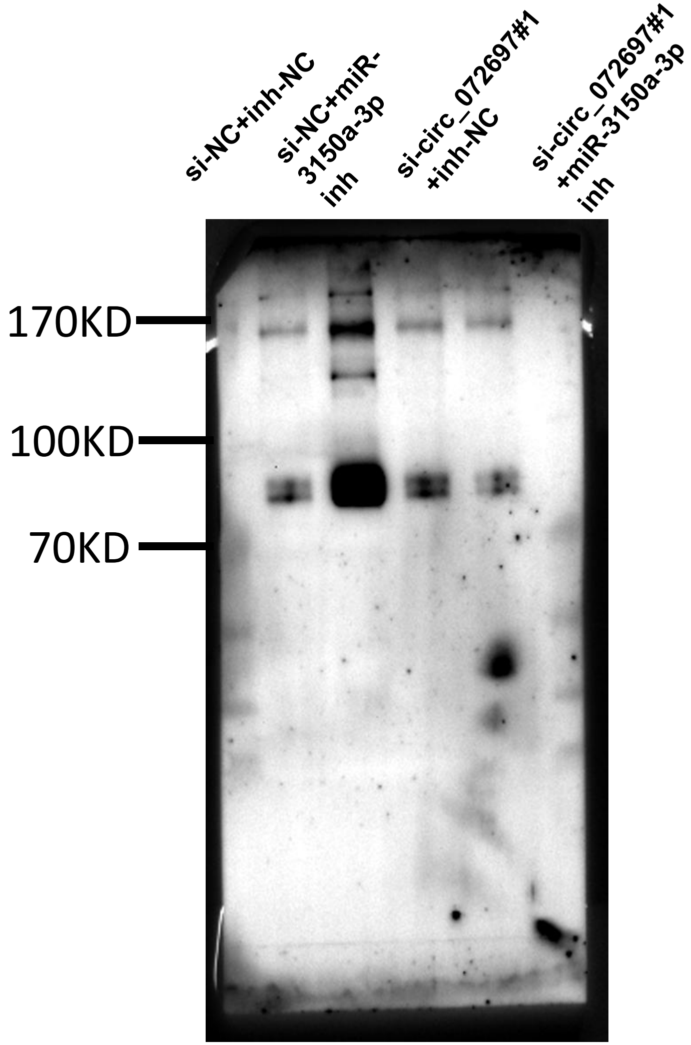

KDM2A

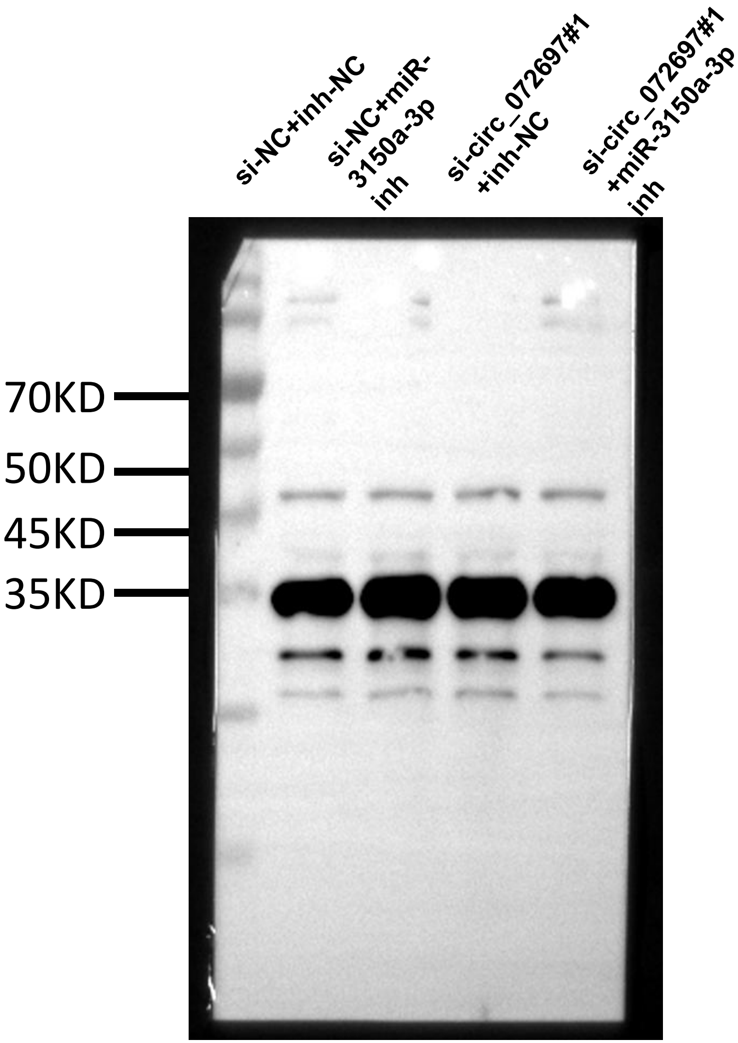

GAPDH
